# Melatonin Plus Folic Acid Treatment Ameliorates Reserpine-Induced Fibromyalgia: An Evaluation of Pain, Oxidative Stress, and Inflammation

**DOI:** 10.3390/antiox8120628

**Published:** 2019-12-06

**Authors:** Roberta Fusco, Rosalba Siracusa, Ramona D’Amico, Alessio Filippo Peritore, Marika Cordaro, Enrico Gugliandolo, Rosalia Crupi, Daniela Impellizzeri, Salvatore Cuzzocrea, Rosanna Di Paola

**Affiliations:** 1 Department of Chemical, Biological, Pharmaceutical and Environmental Sciences, University of Messina, Viale Ferdinando Stagno D’Alcontres, n 31, 98166 Messina, Italy; rfusco@unime.it (R.F.); rsiracusa@unime.it (R.S.); aperitore@unime.it (A.F.P.); cordarom@unime.it (M.C.); egugliandolo@unime.it (E.G.); rcrupi@unime.it (R.C.); dipaolar@unime.it (R.D.P.); 2 Department of Pharmacological and Physiological Science, Saint Louis University School of Medicine, 1402 South Grand Blvd, St. Louis, MO 63104, USA

**Keywords:** fibromyalgia, oxidative stress, pain

## Abstract

Background: Fibromyalgia is a chronic condition characterized by increased sensory perception of pain, neuropathic/neurodegenerative modifications, oxidative, and nitrosative stress. An appropriate therapy is hard to find, and the currently used treatments are able to target only one of these aspects. Methods: The aim of this study is to investigate the beneficial effects of melatonin plus folic acid administration in a rat model of reserpine-induced fibromyalgia. Sprague–Dawley male rats were injected with 1 mg/kg of reserpine for three consecutive days and later administered with melatonin, folic acid, or both for twenty-one days. Results: Administration of reserpine led to a significant decrease in the nociceptive threshold as well as a significant increase in depressive-like symptoms. These behavioral changes were accompanied by increased oxidative and nitrosative stress. Lipid peroxidation was significantly increased, as well as nitrotyrosine and PARP expression, while superoxide dismutase, nonprotein thiols, and catalase were significantly decreased. Endogenously produced oxidants species are responsible for mast cell infiltration, increased expression pro-inflammatory mediators, and microglia activation. Conclusion: Melatonin plus acid folic administration is able to ameliorate the behavioral defects, oxidative and nitrosative stress, mast cell infiltration, inflammatory mediators overexpression, and microglia activation induced by reserpine injection with more efficacy than their separate administration.

## 1. Introduction

Fibromyalgia is a chronic clinical condition characterized by chronic widespread pain, fatigue, depression, and sleep disturbances [1,2,3]. It is widely distributed: 2–5.8% of the population of industrial countries are affected by it [4]. Although aspects of the pathophysiology are still unclear, evidence of involvement of neurotransmitter, genetics, autonomic nervous system (ANS) dysfunction, neuroendocrine dysfunctions, and cerebral psychophysiological abnormalities have been demonstrated [5,6]. Fibromyalgia is considered a non-inflammatory disorder stress-related with dysfunction of the hypothalamic-pituitary-adrenocortical axis [7,8,9]. Furthermore, changes in inflammatory actors [10,11], modified balance in anti- and pro-inflammatory cytokines [12,13], and increases in toxic metabolites of lipid peroxidation and oxidative stress [14,15,16] have been detected. Recent evidences have shown that fibromyalgia syndrome involves the neuropathic pain condition [17]. Hyperalgesia and allodynia are common signs in fibromyalgia [18,19,20]. Sleep deprivation can produce these features [21], in conjunction with inflammation, mitochondrial dysfunction, and oxidative stress, with the result of peripheral nerve damage [22]. Functional brain-imaging studies have displayed compelling evidence for changes in the pain process in fibromyalgia correlating with patients’ allodynia or hyperalgesia [23]. Treatment of fibromyalgia requires a pharmacological approach focused on all symptoms with an emphasis on pain. Several pieces of evidence indicate that melatonin can be useful and suitable in fibromyalgia treatment thanks to its different properties [24,25,26]. It is a highly conserved indoleamine with chronobiological features [27]. Additionally, its anti-inflammatory, antidepressant, analgesic, and sedative activities have been reported [28,29,30,31]. To date, the pathophysiology of the syndrome also shows an important oxidative component [32]. It has long been shown that folic acid can improve the function of the immune system and has important antioxidant properties [33]. It exerts both indirect and direct antioxidant effects, such as protection against oxidative modification of low-density lipoproteins [34], free radical scavenging [35], and activation of cellular antioxidant defense [36,37]. Based on these findings, the aim of this study is to evaluate the effect of folic acid and melatonin administration on a fibromyalgia rat model and compare it with the single administrations of the two substances. 

## 2. Materials and Methods

### 2.1. Animals

Sprague–Dawley male rats (200–230 g, Envigo, Milan, Italy) were used throughout. They received food and *water ad libitum*. The University of Messina Review Board for animal care approved the study. All in vivo experiments followed the new regulations of USA (Animal Welfare Assurance No A5594-01), Europe (EU Directive 2010/63), Italy (D.Lgs 2014/26), and the ARRIVE guidelines.

### 2.2. Induction of Experimental Fibromyalgia

Reserpine administration was performed by subcutaneous injection of 1 mg/kg for three consecutive days [38]. Reserpine (Sigma-Aldrich, Saint Louis, MO, USA) was dissolved in distilled water with 0.5% acetic acid (vehicle). Animals from the sham group received the same volume of vehicle, but they were administered no reserpine.

### 2.3. Experimental Groups

Then, rats were randomly divided into several groups (*n* = 10 for each): 

Group 1. Sham + vehicle: Rats were injected subcutaneously with vehicle (distilled water with a final concentration of 0.5% acetic acid) instead of reserpine and treated orally with saline for 21 days starting from 3 days after first vehicle injection.

Group 2. Sham + melatonin: Rats were injected subcutaneously with vehicle (distilled water with a final concentration of 0.5% acetic acid) instead of reserpine and treated orally with melatonin (10 mg/kg) for 21 days starting from 3 days after first vehicle injection.

Group 3. Sham + folic acid: Rats were injected subcutaneously with vehicle (distilled water with a final concentration of 0.5% acetic acid) instead of reserpine and treated orally with folic acid (3 mg/kg) for 21 days starting from 3 days after first vehicle injection.

Group 4. Sham + melatonin + folic acid (Mel + Fol): Rats were injected subcutaneously with vehicle (distilled water with a final concentration of 0.5% acetic acid) instead of reserpine and treated orally with melatonin (10 mg/kg) and folic acid (3 mg/kg) for 21 days starting from 3 days after first vehicle injection.

Group 5. Reserpine + vehicle: Rats were subjected to injection of reserpine as previously described and treated orally with vehicle (saline) for 21 days starting from 3 days after first reserpine injection.

Group 6. Reserpine + melatonin: Rats were subjected to injection of reserpine as previously described and treated orally with melatonin (10 mg/kg) for 21 days starting from 3 days after first reserpine injection.

Group 7. Reserpine + folic acid: Rats were subjected to injection of reserpine as previously described and treated orally with folic acid (3 mg/kg) for 21 days starting from 3 days after first reserpine injection.

Group 8. Reserpine + melatonin + folic acid (Mel + Fol): Rats were subjected to injection of reserpine as previously described and treated orally with folic acid (3 mg/kg) and melatonin (10 mg/kg) for 21 days starting from 3 days after first reserpine injection.

The dose and route of administration of folic acid and melatonin were chosen based on previous studies [39,40]. Twenty-one days after reserpine injection blood was collected, animals were sacrificed and brain and sciatic nerves were harvested for histological, immunohistochemical and western blot analysis.

### 2.4. Von Frey Hair Test

Mechanical allodynia was evaluated using a dynamic plantar Von Frey hair aesthesiometer on day 0 and 3, 5, 7, 14, and 21 days post-injection (Bio-EVF4; Bioseb, Vitrolles, France) as previously described [41]. The device encloses a force transducer furnished with a plastic tip. When pressure is applied to the tip, the force applied is recorded. The tip was applied to the plantar area of the hind leg, and a rising upward force was exerted until the paw was withdrawn. The withdrawal threshold was defined as the force, expressed in grams, at which the mouse removed the paw.

### 2.5. Hot Plate Test

The hot plate test was performed on day 0 and 3, 5, 7, 14, and 21 days post-injection. The hot-plate latency was evaluated using a metal surface maintained at 53.6 °C (Ugo Basile, Milan, Italy). The rat was monitored and the licking of a hind paw was acquired as the end point. Maximal latency accepted was 45 s [42].

### 2.6. The Tail-Flick Warm Water Test

The tail-flick warm water test was performed on day 0 and 3, 5, 7, 14, and 21 days post-injection. The warm water tail-flick test was employed to evaluate pain threshold. 4 cm of the rat-tail was located in 50 ± 0.5 °C warm water and the time between tail input and retraction was noted (three tests were conducted and the average in units of seconds was recorded). The latency was assessed with a sensitivity of 0.01 s. A maximum tail-flick latency of 10 s was employed to minimize tissue damage to the tail [42].

### 2.7. Forced Swimming Test (FST)

The forced swimming test (FST) was performed on day 0 and 3, 5, 7, 14, and 21 days post-injection according to the original method by Porsolt et al. [43] and modified by Detke and Lucki [44]. Each rat was individually placed in a plexiglass cylinder for 5 min. It was considered immobile when it remained floating in the water making only essential movements to keep its head above water. The total duration of immobility was recorded as immobility time (sec/5 min).

### 2.8. Estimation of Lipid Peroxidation

Twenty-one days after reserpine injection brain tissues were harvested and the malondialdehyde content, an indicator of lipid peroxidation, was measured in the form of thiobarbituric acid-reactive substances by the method of Wills [45]. Briefly, 0.5 mL of cytosolic fraction of brains and 0.5 mL of Tris-HCl were incubated at 37 °C for 2 h. After incubation 1 mL of 10% trichloroacetic acid was added and centrifuged at 1000× *g* for 10 min. Then 1 mL of 0.67% thiobarbituric acid was added to 1 mL of supernatant and the tubes were kept in boiling water for 10 min. After cooling, 1 mL double-distilled water was added and absorbance was measured at 532 nm. Thiobarbituric acid-reactive substances were quantified using an extinction coefficient of 1.56 × 105 M^−1^ cm^−1^ and expressed as nmol of malondialdehyde per mg protein.

### 2.9. Estimation of Non Protein Thiols

Twenty-one days after reserpine injection brain tissues were harvested and non protein thiols were calculated by the method of Jollow [46]. Briefly, 1.0 mL of cytosolic fraction of brain tissues were precipitated with 1.0 mL of sulphosalicylic acid (4%). The samples were kept at 4 °C for at least 1 h and then subjected to centrifugation at 1200× *g* for 15 min at 4 °C. The assay mixture contained 0.1 mL supernatant, 2.7 mL phosphate buffer (0.1 M, pH 7.4) and 0.2 mL 5,5-dithiobis- (2-nitrobenzoic acid) (Ellman’s reagent, 0.1 mM, pH 8.0) in a total volume of 3.0 mL. Samples was read at 412 nm and the reduced glutathione levels were reported as mmol/mg protein.

### 2.10. Estimation of Superoxide Dismutase

Twenty-one days after reserpine injection brain tissues were harvested and superoxide dismutase activity was assayed by the method of Kono [47]. The assay system consisted of 0.1 mM EDTA, 50 mM sodium carbonate, and 96 mM of nitro-blue tetrazolium (NBT). In a cuvette, 2 mL of the above mixture was taken and 0.05 mL of cytosolic fraction of brains and 0.05 mL of hydroxylamine hydrochloride (adjusted to pH 6.0 with NaOH) were added to it. The auto-oxidation of hydroxylamine was observed by measuring the change in optical density at 560 nm for 2 min at 30-/60-s intervals.

### 2.11. Estimation of Catalase

Twenty-one days after reserpine injection brain tissues were harvested and catalase activity was assayed by the method of Claiborne [48]. Briefly, the assay mixture consisted of 1.95 mL phosphate buffer (0.05 M, pH 7.0), 1.0 mL hydrogen peroxide (0.019 M), and 0.05 mL cytosolic fraction of brains in a final volume of 3.0 mL. Changes in absorbance were recorded at 240 nm. Catalase activity was assayed in terms of k min^−1^.

### 2.12. Mast Cells Evaluation

Twenty-one days after reserpine injection brain and sciatic nerve were harvested. Tissues were fixed in 10% buffered formalin, and embedded in paraffin blocks. Seven-μm sections were prepared from paraffin-embedded tissues. After deparaffinization, sections were stained with toluidine blue in order to assess mast cell infiltration. The mast cells count was performed on each slide through a Leica DM6 (Milan, Italy) microscope.

### 2.13. TNF-α and IL-1β ELISA

The quantifications of TNF-α and IL-1β were assayed following the instructions provided by R&D Systems Quantikine Rat TNF- α and IL-1β immunoassay kit [49].

### 2.14. Western Blot Analysis

Western blot analysis was executed on brain and sciatic nerve harvested 21 h after reserpine injection. Cytosolic proteins were extracts as described previously [50]. Membranes were probed with specific Abs: with anti-VEGF (1:500; Santa Cruz Biotechnology, Heidelberg, Germany), or with anti-NGF (1:500; Santa Cruz Biotechnology) in 1× PBS (Phosphate buffered saline), 5% *w*/*v* nonfat dried milk, 0.1% Tween-20 at 4 °C, overnight. To control the equal amounts of proteins, blots also were probed with antibody against b-actin protein (cytosolic fraction 1:500; Santa Cruz Biotechnology). Signals were examined with enhanced chemiluminescence (ECL) detection system reagent according to the manufacturer’s instructions (Thermo Fisher, Waltham, MA, USA). The relative expression of the protein bands was quantified by densitometry with BIORAD ChemiDocTM XRS + software and standardized to b-actin and lamin A/C levels. The blot was stripped with glycine 2% and re-incubated several times to optimize detection of proteins and to visualize other proteins minimizing the number of gels and transfers.

### 2.15. Immunohistochemical Analysis

Immunohistochemical analysis was performed as already described [50]. Tissues were fixed in 10% (*w*/*v*) PBS-buffered formaldehyde and 7 μm sections were prepared from paraffin embedded tissues. After deparaffinization, endogenous peroxidase was quenched with 0.3% (*v*/*v*) hydrogen peroxide in 60% (*v*/*v*) methanol for 30 min. The sections were permeabilized with 0.1% (*w*/*v*) Triton X-100 in PBS for 20 min. Non-specific adsorption was minimized by incubating the section in 2% (*v*/*v*) normal goat serum in PBS for 20 min. Endogenous biotin and avidin binding sites were blocked by sequential incubation for 15 min with biotin and avidin (DBA, Milan, Italy). Subsequently, the sections were incubated overnight with: anti-nitrotyrosine antibody (1:100; Millipore, Abingdon, UK) or anti-PARP antibody (1:100; Santa Cruz Biotechnology). Sections were washed with PBS and incubated with peroxidase-conjugated bovine anti-mouse IgG, secondary antibody (1:2000 Jackson Immuno Research, WestGrove, Pennsylvania, USA). Specific labeling was provided with a biotin-conjugated goat anti-mouse IgG and avidin-biotin peroxidase complex (Vector Laboratories, Burlingame, California, USA). Images were collected using a Leica DM6 (Milan, Italy) microscope. The percentage area of immunoreactivity (described by the number of positive pixels) was reported as percent of total tissue area (red staining).

### 2.16. Immunofluorescence Analysis

Brain sections were incubated with primary antibodies: anti-CD11b (1:100, abcam) or anti Iba-1 (1:100, Santa Cruz Biotechnology) in a humidified chamber at 37 °C overnight. Sections were washed with PBS and were incubated with secondary antibody FITC-conjugated anti-mouse Alexa Fluor-488 antibody (1:2000 *v*/*v* Molecular Probes, UK) for 1 h at 37 °C. Sections were laved and for nuclear staining 4′,6′-diamidino-2-phenylindole (DAPI; Hoechst, Frankfurt; Germany) 2 μg/mL in PBS was added. Sections were analysed using a Leica DM2000 microscope [51]. 

### 2.17. Materials

All compounds used in this study, except where differently specified, were purchased from Sigma-Aldrich Company Ltd.

### 2.18. Statistical Evaluation

All values in the figures and text are expressed as mean ± standard error of the mean (SEM) of N number of animals. In those experiments involving histology, the exhibited pictures are representative of at least three experiments performed on different days. Results were analyzed by one-way ANOVA followed by a Bonferroni post-hoc test for multiple comparisons. A *p*-value <0.05 was considered significant. * *p* < 0.05 vs. sham, ° *p* < 0.05 vs. vehicle, ** *p* < 0.01 vs. sham, °° *p* < 0.01 vs. vehicle, *** *p* < 0.001 vs. sham, °°° *p* < 0.001 vs. vehicle.

## 3. Results

### 3.1. Effect of Folic Acid and Melatonin Treatment on Behavioral Defects Induced by Reserpine Injection

**Figure 1:**
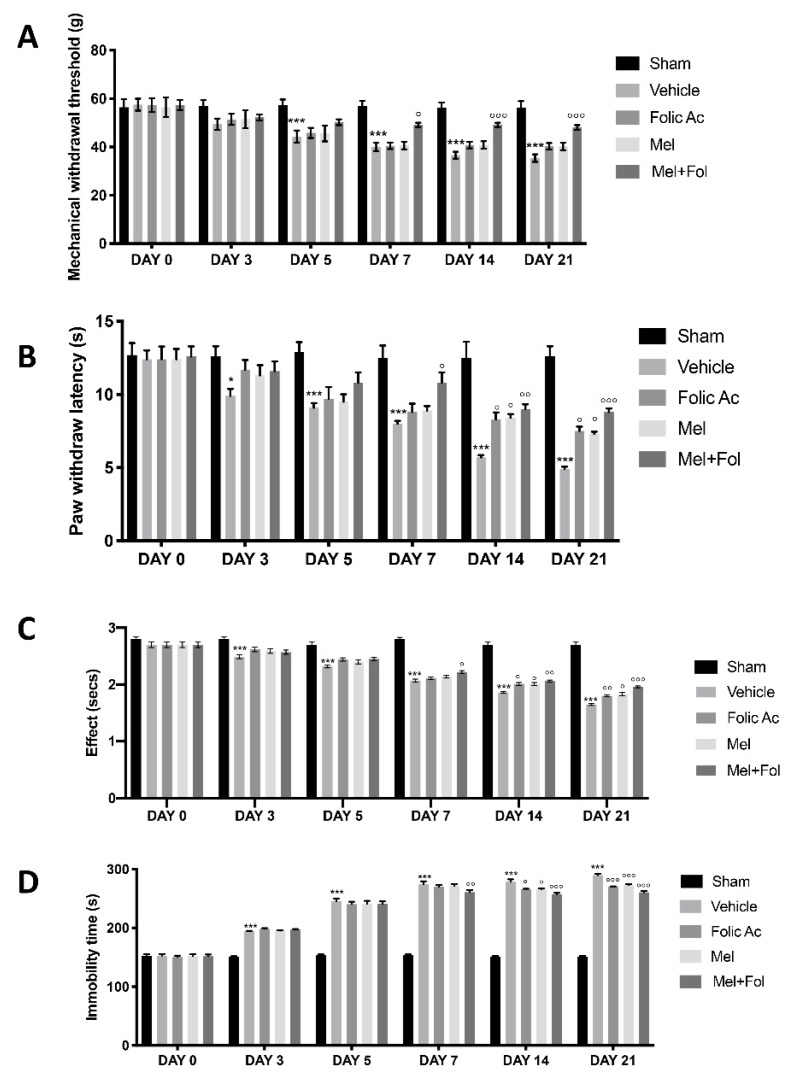
Efficacy of folic acid and melatonin administration on behavioral changes reserpine-induced. Behavioral tests: (**A**) Von Frey test, (**B**) hot plate test, (**C**) tail-flick test, (**D**) forced swimming test (D). A *p*-value < 0.05 was considered significant. * *p* < 0.05 vs. sham, ° *p* < 0.05 vs. vehicle, °° *p* < 0.01 vs. vehicle, *** *p* < 0.001 vs. sham, °°° *p* < 0.001 vs. vehicle.

Mechanical hyperalgesia was evaluated by a von Frey test. Reserpine injection produced a significant decrease in paw-withdrawal threshold in response to von-Frey hair stimulation in vehicle treated rats compared to sham groups (Figure S1). Mel + Fol treatment significantly increased the paw-withdrawal threshold in reserpine-treated rats, compared to melatonin and folic acid (Figure 1A). In addition, the effect of Mel + Fol treatment on pain sensitivity was tested by subjecting rats to hot plate and tail-flick tests. Reserpine injection produced an increased pain sensitivity in vehicle group compared to control groups (Figure S1). Mel + Fol treatment displayed an antinociceptive effect in hot plate (Figure 1B) and tail-flick tests (Figure 1C) in reserpine-treated rats, compared to melatonin and folic acid. The depressive-like behavior was evaluated by the forced swimming test. Reserpine injection increased the immobility time in reserpine-vehicle treated animals, compared to the sham groups (Figure S1). Mel + Fol treatment significantly decreased the immobility time in reserpine-treated rats, compared to melatonin and folic acid (Figure 1D).

### 3.2. Effect of Folic Acid and Melatonin Treatment on Lipid Peroxidation and Anti-Oxidant Profile Induced by Reserpine Injection

**Figure 2:**
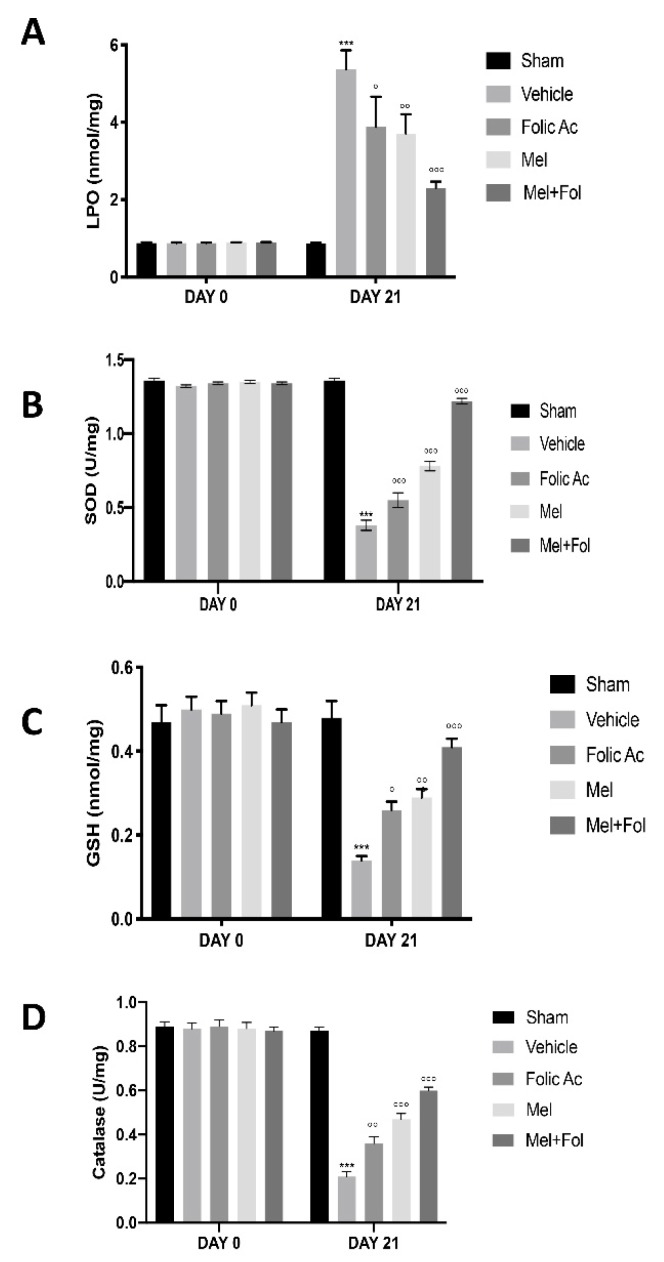
Efficacy of folic acid and melatonin administration on oxidative stress reserpine-induced. (**A**) Estimation of lipid peroxidation, (**B**) estimation of non protein thiols, (**C**) estimation of superoxide dismutase, (**D**) estimation of catalase. A *p*-value < 0.05 was considered significant. ° *p* < 0.05 vs. vehicle, °° *p* < 0.01 vs. vehicle, *** *p* < 0.001 vs. sham, °°° *p* < 0.001 vs. vehicle.

It has been shown that oxidative stress is implicated in the pathogenesis of fibromyalgia [16]. Lipid peroxide levels were increased in reserpine-vehicle treated rats compared to sham groups (Figure S2). Treatment with Mel + Fol caused a significant reduction in lipid peroxide in reserpine-treated rats, compared to melatonin and folic acid (Figure 2A). The enzymatic activity of superoxide dismutase (Figure 2B), non-protein thiols (Figure 2C), and catalase (Figure 2D) significantly decreased in the reserpine-vehicle treated rats compared to the sham groups (Figure S2). This reduction was significantly restored with in animals treated with Mel + Fol, compared to melatonin and folic acid.

### 3.3. Effect of Folic Acid and Melatonin Treatment on Nitrosative Stress and PARP Expression Induced by Reserpine Injection

**Figure 3:**
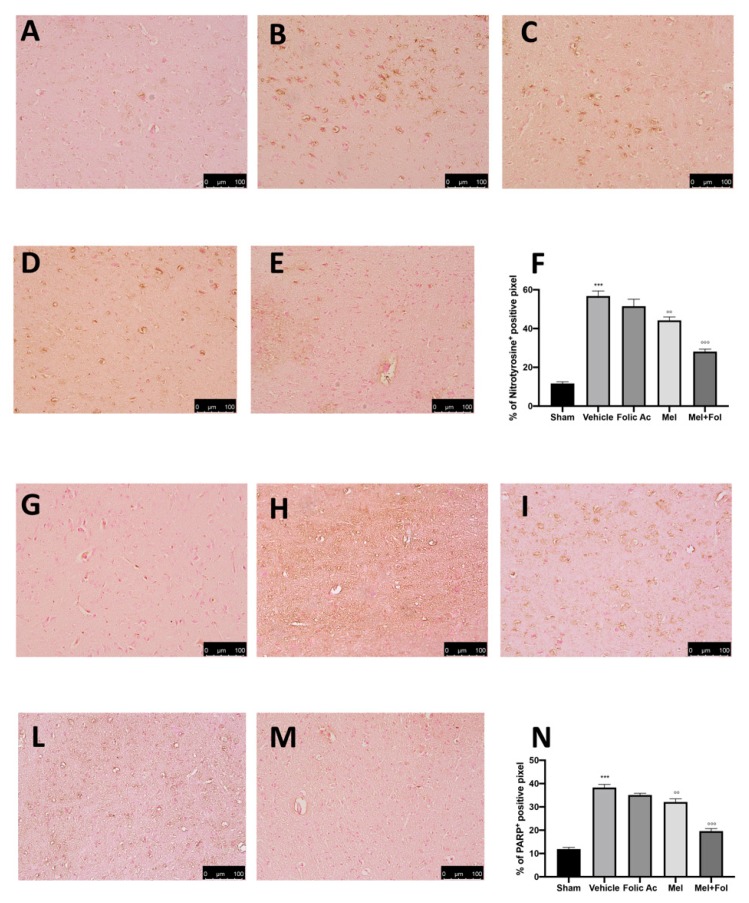
Efficacy of folic acid and melatonin administration on nitrityrosine and PARP expression reserpine-induced. Immunohistochemistry evaluation of nitrityrosine expression in (**A**) sham, (**B**) vehicle, (**C**) folic acid, (**D**) melatonin, (**E**) melatonin plus folic acid and (**F**) graphical quantification. Immunohistochemistry evaluation of PARP expression in (**G**) sham, (**H**) vehicle, (**I**) folic acid, (**L**) melatonin, (**M**) melatonin plus folic acid, and (**N**) graphical quantification. A *p*-value < 0.05 was considered significant, °° *p* < 0.01 vs. vehicle, *** *p* < 0.001 vs. sham, °°° *p* < 0.001 vs. vehicle.

Twenty-one days after reserpine injection, we also investigated nitrotyrosine and PARP expression associated with oxidative stress by immunohistochemistry. Increased nitrotyrosine and PARP expression was found in brain tissue sections of reserpine-vehicle treated (Figure 3B,F,H,N) rats compared with the sham groups (Figure 3A,F,G,N and Figure S3). Treatment with Mel + Fol caused a significant reduction in nitrotyrosine (Figure 3E,F) and PARP expression (Figure 3M,N) in reserpine-treated rats, compared to melatonin (Figure 3D,F,L,N) and folic acid (Figure 3C,F,I,N).

### 3.4. Effect of Folic Acid and Melatonin Treatment on Mast Cells Infiltration induced by Reserpine Injection

**Figure 4:**
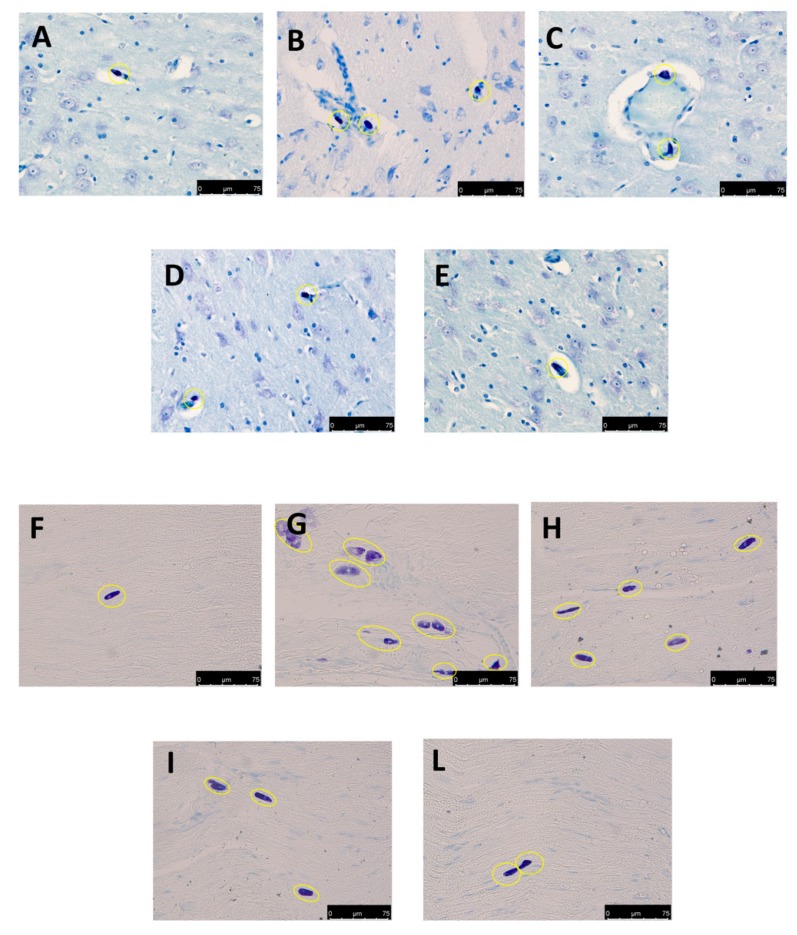
Efficacy of folic acid and melatonin administration on mast cells activation reserpine-induced. Evaluation of mast cell degranulation by toluidine blue in brain: (**A**) sham, (**B**) vehicle, (**C**) folic acid, (**D**) melatonin, (**E**) melatonin plus folic acid. Evaluation of mast cell degranulation by toluidine blue in sciatic nerve: (**F**) sham, (**G**) vehicle, (**H**) folic acid, (**I**) melatonin, (**L**) melatonin plus folic acid. 40× magnification is shown.

Twenty-one days after reserpine injection, mast cells infiltration and degranulation were assessed by toluidine blue staining. There was a significant up-regulation in mast cell number, which performs a key role in the development of hyperalgesia and in the inflammatory process, both in brain (Figure 4B) and sciatic nerve (Figure 4G) in reserpine-vehicle treated rats, compared to the sham groups (Figure 4A,F and Figure S4). Mel + Fol treatment reduced the number of mast cells both in brain (Figure 4E) and sciatic nerve (Figure 4L) in reserpine-treated rats, more than melatonin (Figure 4D,I) and folic acid (Figure 4C,H).

### 3.5. Effect of Folic acid and Melatonin Treatment on Changes in Pro-Inflammatory, Vasoactive and Neuro-Sensitizing Mediators Induced by Reserpine Injection

**Figure 5:**
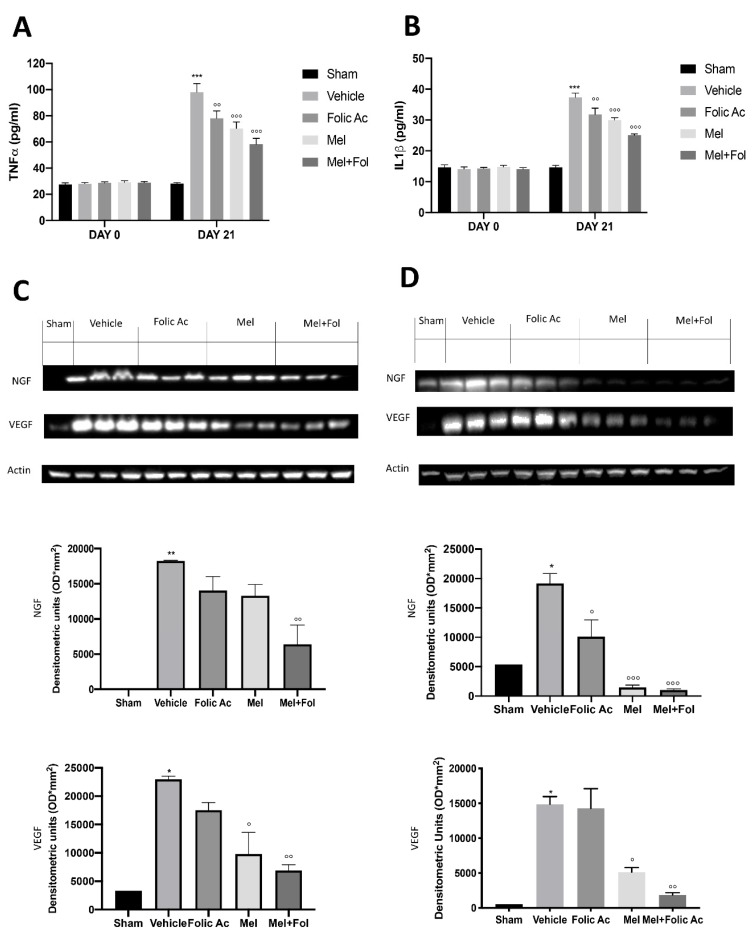
Efficacy of folic acid and melatonin administration on IL-1β, TNF-α, VEGF, and NGF expression reserpine-induced. Elisa kit of (**A**) IL-1β and (**B**) TNF-α levels. Western blots and respectively quantification of VEGF and NGF in (**C**) brain and (**D**) sciatic nerve. A *p*-value < 0.05 was considered significant. * *p* < 0.05 vs. sham, ° *p* < 0.05 vs. vehicle, ** *p* < 0.01 vs. sham, °° *p* < 0.01 vs. vehicle, *** *p* < 0.001 vs. sham, °°° *p* < 0.001 vs. vehicle.

Twenty-one days after reserpine injection, IL-1β and TNF-α levels were increased in reserpine-vehicle treated rats, compared to the sham groups (Figure S5). Treatment with Mel + Fol produced a significant reduction in IL-1β (Figure 5A) and TNF-α (Figure 5B) levels in reserpine-treated rats, compared to melatonin and folic acid. Western blot analysis showed NGF and VEGF increased expression in both brain and nerve tissues harvested from reserpine-vehicle treated animals, compared to the sham groups (Figure 5C,D and Figure S5). Mel + Fol administration significantly reduced NGF and VEGF expression in reserpine-treated rats with more efficacy than melatonin and folic acid (Figure 5C,D).

### 3.6. Effect of Folic Acid and Melatonin Treatment on Microglia Activation Induced by Reserpine Injection

**Figure 6:**
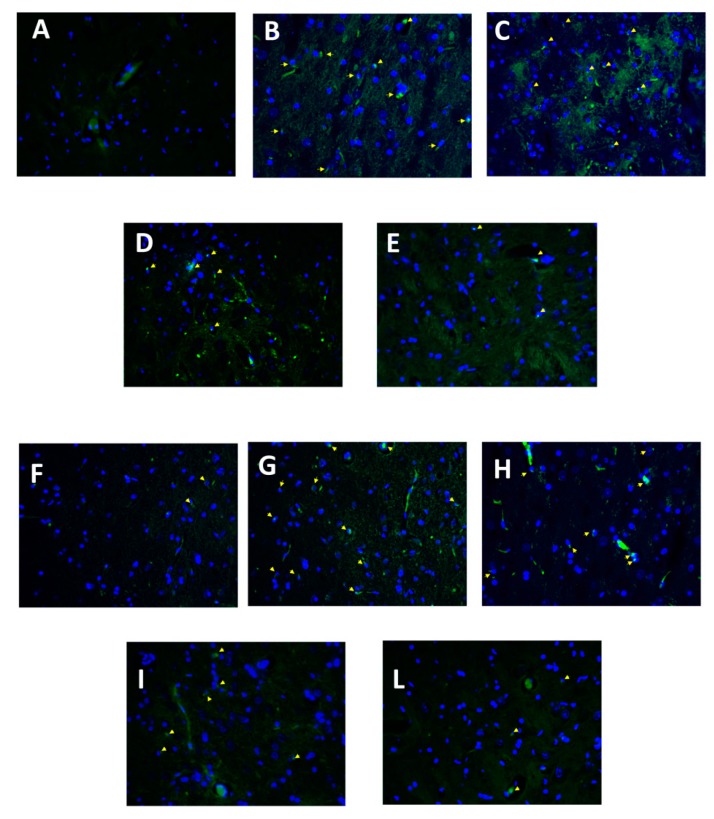
Efficacy of folic acid and melatonin administration on Iba1 and CD11b expression reserpine-induced. Immunofluorescence of brain Iba1 in (**A**) sham, (**B**) vehicle, (**C**) folic acid, (**D**) melatonin, (**E**) melatonin plus folic acid. Immunofluorescence of brain CD11b in (**F**) sham, (**G**) vehicle, (**H**) folic acid, (**I**) melatonin, (**L**) melatonin plus folic acid. 40× magnification is shown.

Twenty-one days after reserpine injection, we also investigated microglial activation by immunofluorescence. Increased Iba1 and CD11b positive cells were found in brain tissue sections of reserpine-vehicle treated (Figure 6B,G) rats compared with the sham groups (Figure 6A,F and Figure S6). Treatment with Mel + Fol caused a significant reduction in Iba1 (Figure 6E) and Cd11b positive cells (Figure 6L) in reserpine-treated rats, compared to melatonin (Figure 6D,I) and folic acid (Figure 6C,H).

## 4. Discussion

Fibromyalgia is a multisymptomatic and multifactorial disease [20]. The pathophysiological mechanisms by which the disease is characterize include, among others, changes in sensory perception of pain [52], oxidative stress and inflammation [18] with damage to myelinated and nonmyelinated nerve fibers [16]. In our study, animals subjected to fibromyalgia showed increased pain sensitivity in mechanical allodynia and thermal hyperalgesia. Moreover, this enhanced sensibility was coupled with depression symptoms, as indicated by the rat behaviour in the forced swim test. Several evidence, in fact, indicates that the depression-like symptoms in rats increased allodynia and hyperalgesia under the condition of fibromyalgia [3]. A concomitant treatment of melatonin and folic acid was able to reduce the increased pain sensibility and the depression-like behaviour with more efficacy than them single administration. Increasing evidence suggests that enhanced oxidative stress and nitric oxide are involved in the fibromyalgia pathophysiology and increase the severity of the symptoms [53,54]. We are in line with literature [55,56]; our data also underlines that the oxidative and nitrosative stress induces neurogenic inflammation which is responsible for the perpetuation of pain [16]. The oxidants and antioxidants equilibrium is unbalanced in this pathology [32]: increased lipid peroxidation was detected in rats subjected to fibromyalgia, while superoxide dismutase, nonprotein thiols and catalase were significantly decreased [49]. Thanks to its antioxidant properties, a combined treatment of melatonin and folic acid was also able to reduce these parameters better than the single administration. While free oxygen radicals oxidize membrane phospholipids amplifying lipid peroxidation, nitric oxide excessively produced by iNOS reacts with superoxide anions yielding the toxic oxidizing agent peroxynitrite. It nitrates tyrosine residues, causing changes in protein function and structure that induce tissue damage. Peroxynitrite in turn activates PARP, a single-strand break DNA repair enzyme that acts by synthesizing chains of ADP-ribose [50]. To product ADP-ribose monomers, the obligate substrate is NAD+. PARP hyper-activation depletes NAD+ cellular reserves of leading to ATP depletion, cellular dysfunction, and death. Melatonin plus folic acid administration decreased nitrotyrosine and PARP staining induced by fibromyalgia better than the single administration of the two substances. This increased oxidative stress is able to induce mast cells activation [57]. Systemic mastocytosis [58] is commonly experience in patients affected by fibromyalgia [59,60]. Several pieces of evidence show the importance of mast cells activation in this disease and [61,62] comorbid disorders [63] such as neuroimmune interactions [64] and painful conditions, [65,66]. Mast cells reside near the nerve fibers, which give them the possibility to migrate for modulating nociception and neural activity [59,67,68,69]. As result of their migration and degranulation, there is an important release of pro-inflammatory, vasoactive and neuro-sensitizing mediators [70]. In particular, an increased expression of cytokines (IL-1β and TNF-α) [71,72] and growth factors (NGF and VEGF) [73,74,75] that contribute to the maintenance of inflammation and pain have been detected in both nerve and brain [76,77,78,79]. An associate administration of melatonin and folic acid was able to decrease mast cells infiltration and the related increased expression of pro-inflammatory cytokines and vasoactive and neuro-sensitizing mediators with more efficacy than them single administration. Mast cells also communicate with microglia [60,80,81]. In the contests of pain, microglia in the thalamus is responsible for maintaining the pain sensation even after the original stimulus is over [82,83]. The concomitant treatment of melatonin and folic acid was able to reduce the increased microglia activation, assessed by Iba1 and CD11b expression, with more efficacy than their single administration.

## 5. Conclusions

Our results provide evidence that a combined treatment of melatonin and folic acid may be useful in the treatment of fibromyalgia, thanks to its ability to target all mediators that contribute to the perpetuation of pain, from the mastocytosis and related pro-inflammatory, vasoactive and neuro-sensitizing mediators to the oxidative stress processes.

## Supplementary Materials

The following are available online at https://www.mdpi.com/2076-3921/8/12/628/s1, Figure S1: Efficacy of folic acid and melatonin administration on behavioral changes of sham groups, Figure S2: Efficacy of folic acid and melatonin administration on oxidative stress of sham groups, Figure S3: Efficacy of folic acid and melatonin administration on nitrityrosine and PARP expression of sham groups, Figure S4: Efficacy of folic acid and melatonin administration on mast cells activation of sham groups, Figure S5: Efficacy of folic acid and melatonin administration on IL-1β, TNF-α, VEGF and NGF expression of sham groups, Figure S6: Efficacy of folic acid and melatonin administration on Iba1 and CD11b expression of sham groups.
