# Supplementary material for: Melatonin Plus Folic Acid Treatment Ameliorates Reserpine-Induced Fibromyalgia: An Evaluation of Pain, Oxidative Stress, and Inflammation
Source: Antioxidants (Basel). 2019 Dec 6;8(12):628. doi: 10.3390/antiox8120628 (PMC6943570; doi:10.3390/antiox8120628)
Supplement: Supplementary file 1 [file antioxidants-08-00628-s001.pdf]

Supplementary Materials:

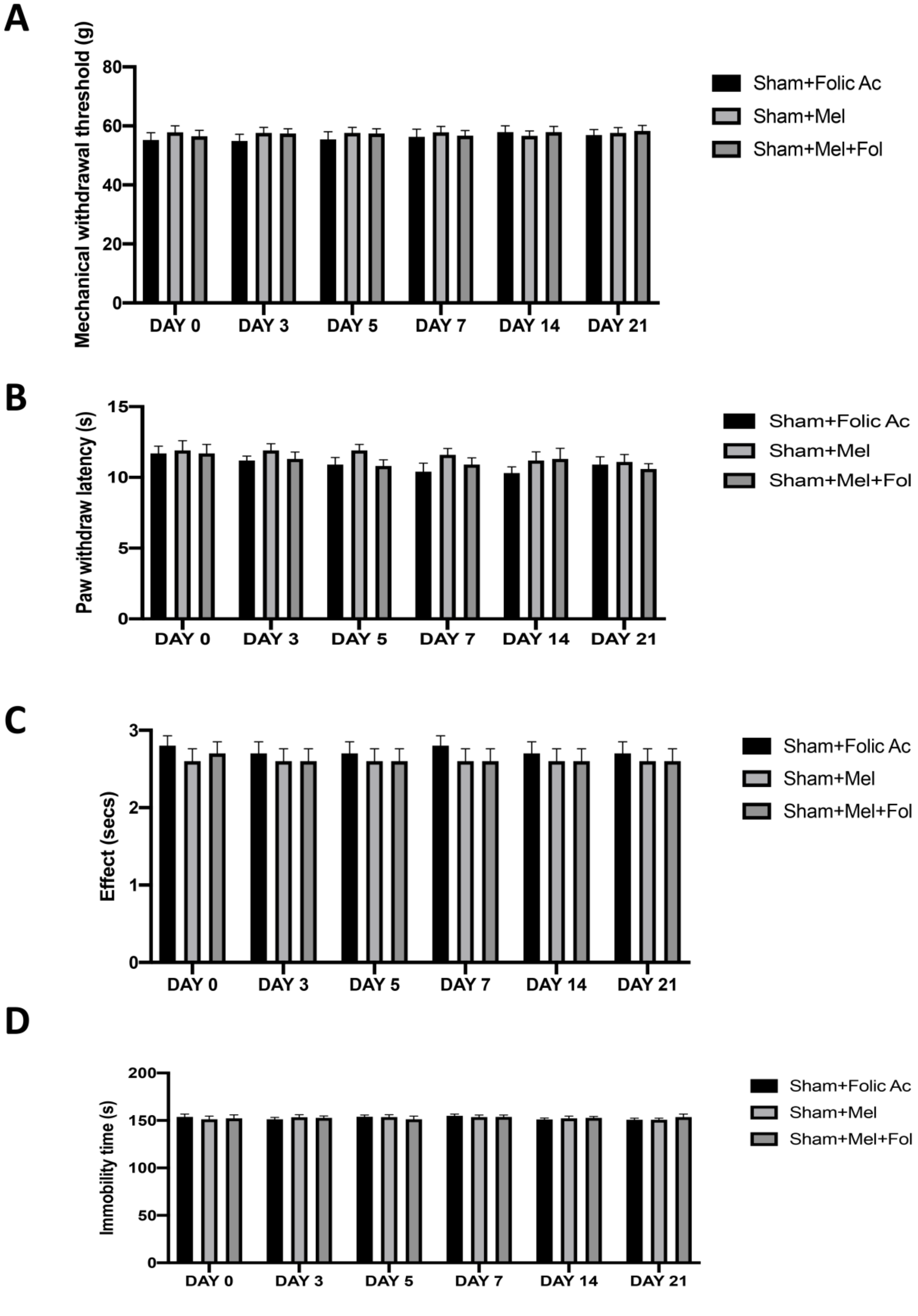

Figure S1. Efficacy of folic acid and melatonin administration on behavioral changes of sham groups. Behavioral tests: (**a**)Von Frey test, (**b**) Hot Plate test, (**c**) Tail-flick test, (**d**) Forced Swimming test (D).

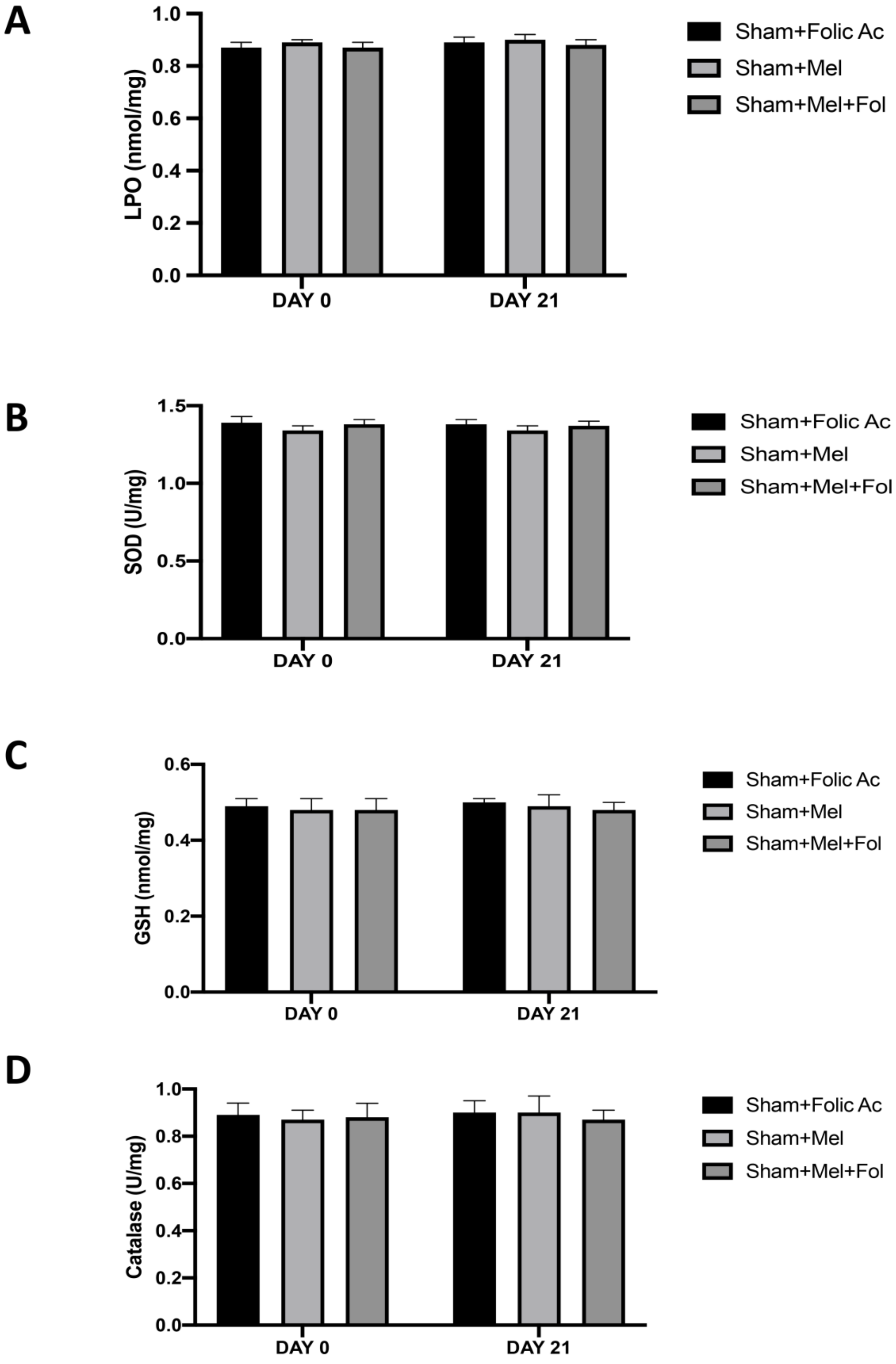

Figure S2. Efficacy of folic acid and melatonin administration on oxidative stress of sham groups. (a) Estimation of lipid peroxidation, (b) Estimation of non protein thiols, (c) Estimation of superoxide dismutase, (d) Estimation of catalase.

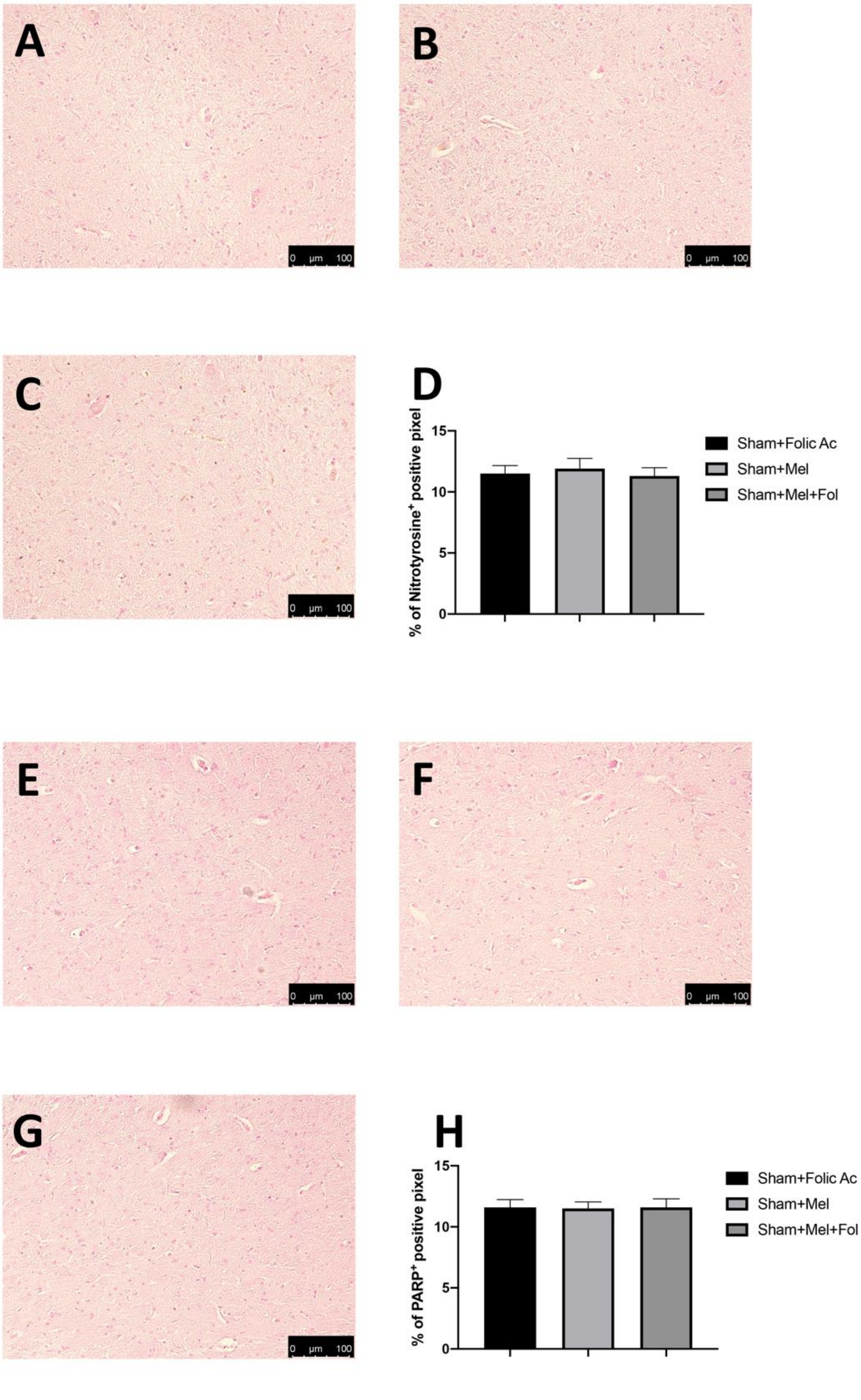

Figure S3. Efficacy of folic acid and melatonin administration on nitrityrosine and PARP expression of sham groups. Immunohistochemistry evaluation of nitrityrosine expression in (a) sham + folic acid, (b) sham + melatonin, (c) sham + melatonin plus folic acid, (d) graphical quantification. Immunohistochemistry evaluation of PARP expression in (e) sham + folic acid, (f) sham + melatonin, (g) sham + melatonin plus folic acid, (h) graphical quantification.

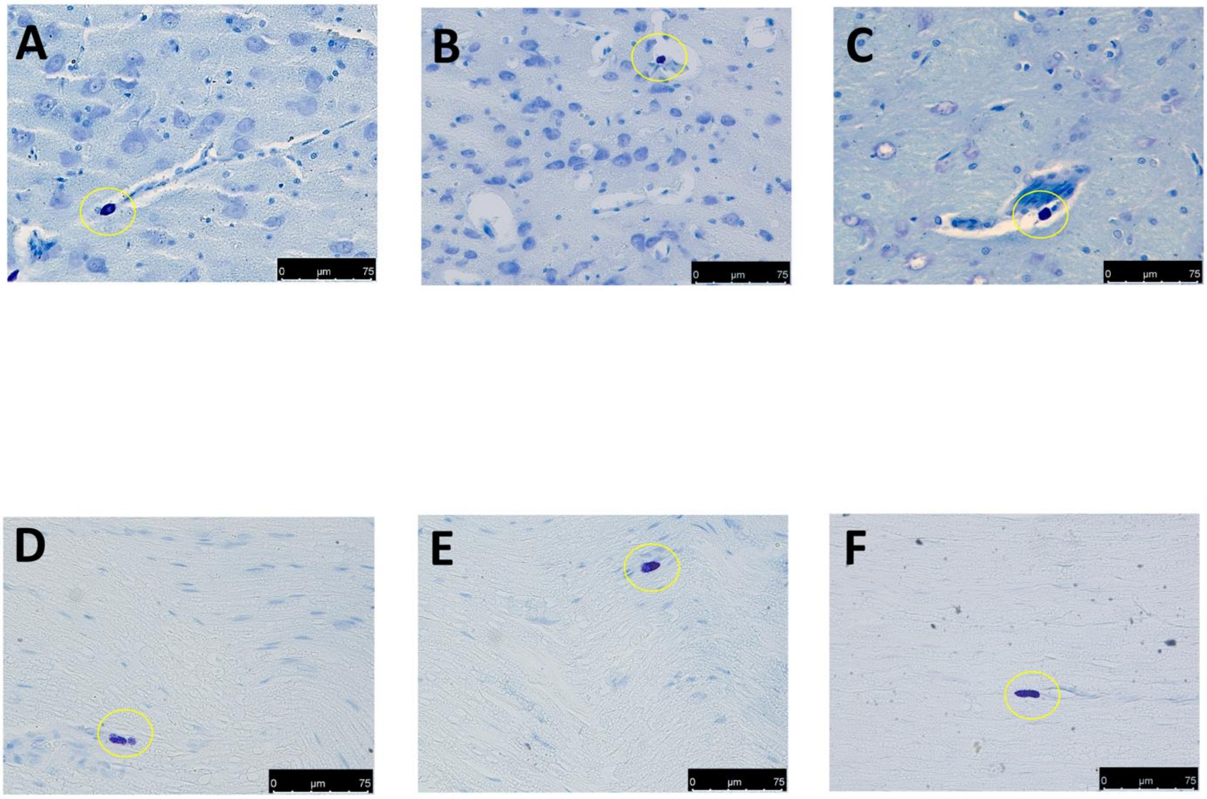

Figure S4. Efficacy of folic acid and melatonin administration on mast cells activation of sham groups. Evaluation of mast cell degranulation by toluidine blue in brain: (a) sham + folic acid, (b) sham + melatonin, (c) sham + melatonin plus folic acid. Evaluation of mast cell degranulation by toluidine blue in sciatic nerve: (d) sham + folic acid, (e) sham + melatonin, (f) sham + melatonin plus folic acid.

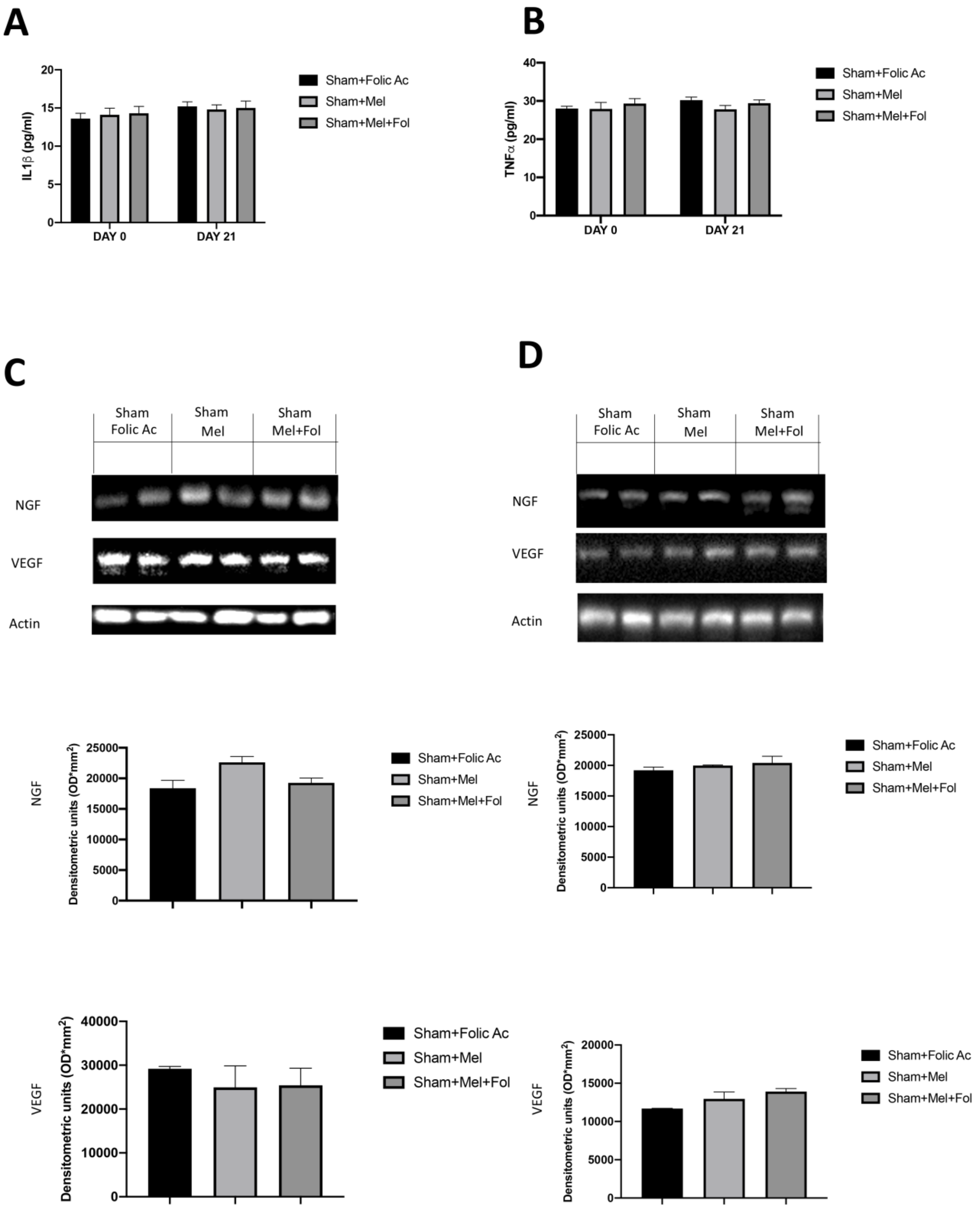

Figure S5. Efficacy of folic acid and melatonin administration on IL-1β, TNF-α, VEGF and NGF expression of sham groups. Elisa kit of (a) IL-1β and (b) TNF-α levels. Western blots and respectively quantification of VEGF and NGF in (c) brain and (d) sciatic nerve.

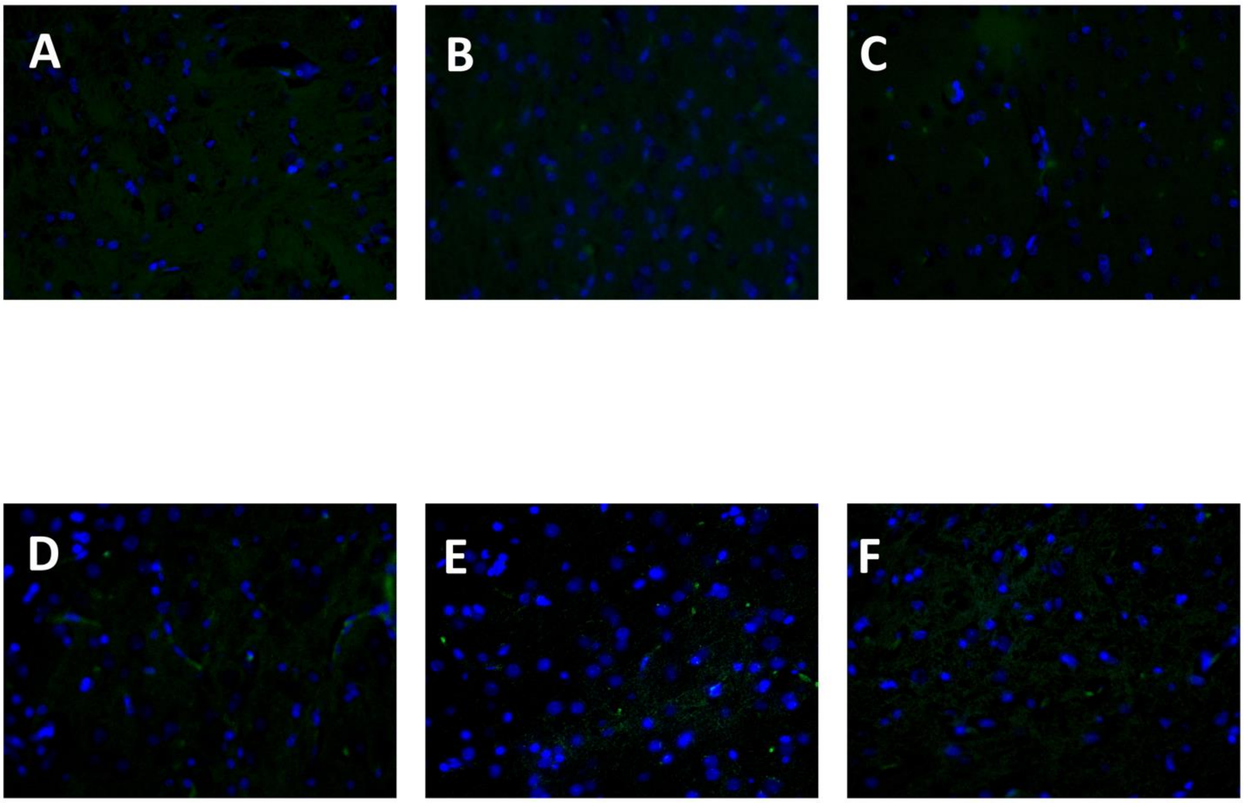

Figure S6. Efficacy of folic acid and melatonin administration on Iba1 and CD11b expression of sham groups. Immunofluorescence of brain Iba1 in (a) sham + folic acid, (b) sham + melatonin, (c) sham + melatonin plus folic acid. Immunofluorescence of brain CD11b in (d) sham + folic acid, (e) sham + melatonin, (f) sham + melatonin plus folic acid.
